# Monopolar Capacitive-Resistive Radiofrequency for Pelvic Floor Hypertonia and Vaginismus: A Case Series

**DOI:** 10.7759/cureus.115396

**Published:** 2026-08-29

**Authors:** Pablo González-Isaza, Diana Vélez, Manuel Sánchez-Prieto, Manuela Cerón, Rafael Sánchez-Borrego, Anibal Nieto Diaz

**Affiliations:** 1 Department of Urogynecology, San Jorge University Hospital, Pereira, COL; 2 Department of Urogynecology, Soranus Center for Aesthetic and Functional Urogynecology, Pereira, COL; 3 Department of Obstetrics and Gynecology, Instituto Universitario Dexeus, Barcelona, ESP; 4 Department of Obstetrics and Gynecology, DIATROS, Clínica de Atención a la Mujer, Barcelona, ESP; 5 Department of Obstetrics and Gynecology, Clinic Universitary La Arrixaca Hospital, Murcia, ESP

**Keywords:** chronic pelvic pain syndrome, female sexual function, pelvic floor hypertonia, pelvic floor myofascial pain syndrome, radiofrequency therapy, vaginismus

## Abstract

Pelvic floor hypertonia is an important musculoskeletal contributor to chronic pelvic pain and may be associated with genito-pelvic pain/penetration disorders, including vaginismus. We describe a prospective case series of three women aged 31, 32, and 45 years with pelvic floor hypertonia and vaginismus who underwent six weekly treatment sessions using a monopolar capacitive-resistive radiofrequency device (C200; Capenergy Medical, S.L., Sant Joan Despí, Barcelona, Spain). Each session consisted of a 20-minute intracavitary application using the MJS (Medical Jet System) handpiece combined with simultaneous application of an automatic pelvic plate. Outcomes were assessed before treatment and at follow-up using pain scores, the Female Sexual Function Index (FSFI), pelvic floor examination, and functional assessment of vaginal penetration. Baseline pain scores ranged from 8 to 9 out of 10 and decreased to 0 to 1 at follow-up. FSFI total scores increased from a baseline range of 8.3-12.4 to 28.9-31.8 at follow-up. All three patients showed improvement in clinically assessed pelvic floor muscle hypertonia and regained pain-free vaginal penetration, with satisfactory penetrative intercourse reported during follow-up. In this small uncontrolled case series, monopolar capacitive-resistive radiofrequency treatment was associated with clinically relevant improvements in pelvic pain, pelvic floor examination findings, and sexual function. These findings are preliminary and hypothesis-generating and cannot establish treatment efficacy. Larger controlled prospective studies using standardized treatment protocols, objective pelvic floor assessments, and predefined long-term follow-up are needed to determine the efficacy, safety, and appropriate clinical role of this intervention.

## Introduction

Chronic pelvic pain is a complex condition with gynecologic, urologic, gastrointestinal, musculoskeletal, and neurologic contributors and can substantially impair physical, sexual, and psychosocial well-being. Among its musculoskeletal components, pelvic floor hypertonia is characterized by increased resting activity or impaired relaxation of the pelvic floor musculature and may contribute to pelvic pain, dyspareunia, urinary and defecatory symptoms, and sexual dysfunction [1].

Pelvic floor hypertonia may also play an important role in genito-pelvic pain/penetration disorders, including vaginismus [2]. These conditions involve persistent difficulties with vaginal penetration that may arise from interacting muscular, nociceptive, behavioral, and psychosexual factors. Accordingly, management is typically individualized and multidisciplinary, with pelvic floor physical therapy, manual and myofascial techniques, relaxation and breathing strategies, vaginal dilators, and psychosexual interventions forming important components of conservative treatment [2]. Nevertheless, some patients experience persistent or recurrent symptoms despite these approaches.

Radiofrequency-based therapies have therefore been explored as adjunctive modalities for myofascial pelvic pain and pelvic floor dysfunction. Capacitive-resistive radiofrequency transfers electromagnetic energy to tissues, producing controlled heating that may increase local perfusion, influence muscle relaxation, and modulate pain-related mechanisms. Preliminary clinical studies, including controlled trials, have reported reductions in pelvic pain and improvements in pelvic floor-related outcomes following radiofrequency-based interventions [3-6]. However, available evidence remains limited, treatment protocols and technologies are heterogeneous, and data specifically addressing pelvic floor hypertonia associated with vaginismus are scarce.

The C200 device (Capenergy Medical, S.L., Sant Joan Despí, Barcelona, Spain) is a monopolar capacitive-resistive radiofrequency platform that allows intracavitary treatment combined with an external automatic pelvic plate. Given the limited evidence regarding this approach in women with pelvic floor hypertonia and vaginismus, we describe the clinical course and patient-reported outcomes of three women treated with a six-session C200 protocol, focusing on changes in pelvic pain, sexual function, pelvic floor examination findings, and vaginal penetration.

## Case presentation

Patient selection and treatment protocol

This prospective case series included three women with chronic pelvic pain associated with pelvic floor hypertonia and vaginismus who underwent treatment with the C200 monopolar capacitive-resistive radiofrequency device (Capenergy Medical, S.L., Sant Joan Despí, Barcelona, Spain). All patients provided written informed consent before treatment and for the use of their anonymized clinical data for scientific publication.

Patients were considered eligible when pelvic floor hypertonia had been clinically identified as a relevant contributor to pelvic pain or penetration difficulty, symptoms had persisted for more than six months, baseline pain intensity was greater than four on a 10-point Visual Analog Scale (VAS), and symptoms adversely affected sexual function or quality of life. Exclusion criteria included pregnancy, active pelvic infection, active malignancy, an implanted pacemaker or other electronic device, and pelvic surgery within the preceding six months.

Pelvic floor assessment was performed by vaginal examination and included evaluation of resting muscle tone, ability to relax the pelvic floor, tenderness to palpation, and clinically evident contracture of the levator ani, puborectalis, and transverse perineal muscles, as appropriate to each patient. Functional tolerance of vaginal penetration and, when relevant, vaginal dilator use were also documented.

All patients underwent six treatment sessions at one-week intervals. Each session consisted of a 20-minute intracavitary application using the Medical Jet System (MJS) handpiece combined with simultaneous 20-minute application of an automatic pelvic plate. Treatment was administered by personnel trained in the use of the device. Detailed session-level energy and temperature parameters were not systematically recorded. No tissue ablation was intended.

Clinical outcome measures were recorded at baseline and reassessed after completion of the six-session protocol and at subsequent follow-up. Pain was evaluated using the 10-point VAS, and sexual function was assessed with the Female Sexual Function Index (FSFI) [7]. Pelvic floor examination findings and functional vaginal penetration were documented clinically. As this was an exploratory three-patient case series, outcomes were reported descriptively, and no inferential statistical analyses were performed.

Baseline clinical characteristics, previous treatments, pelvic floor examination findings, and patient-reported outcome scores are summarized in Table 1.

**Table 1:** Baseline clinical characteristics of the three patients FSFI: Female Sexual Function Index.

Characteristics	Case 1	Case 2	Case 3
Age, years	32	31	45
Main clinical presentation	Recurrent vaginismus	Vaginismus with marked apprehension toward vaginal penetration	Vaginismus with severe deep dyspareunia
Pelvic floor hypertonia	Present	Present	Present
Relevant medical history	Migraine	Bipolar affective disorder; personality disorder	Hypothyroidism; hysterectomy (2023); biopsy-confirmed vulvar lichen sclerosus
Symptom history	Recurrent symptoms after discontinuation of vaginal dilators for >1 year	Persistent penetration-related symptoms with fear and anticipatory anxiety	Approximately 10 years of vulvovaginal symptoms following childbirth
Previous pelvic floor physical therapy	Not reported	Yes; intermittently interrupted	Yes
Previous vaginal dilator therapy	Yes; partial previous improvement	Yes; limited adherence	Yes
Other relevant previous treatment	None reported	None reported	High-potency topical corticosteroids
Baseline pelvic floor examination	Marked contracture of levator ani, puborectalis, and transverse perineal muscles	Bilateral levator ani contracture; moderate-to-severe pain on examination; introital tenderness	Increased pelvic floor muscle tone; pain on palpation of anterior vaginal wall, levator ani, and transverse perineal muscles
Additional baseline vulvovaginal findings	Mild erythema and irritation of left labium majus	None reported	Clitoral hood phimosis; labial architectural changes; pale and dry vaginal mucosa
Baseline pain score, 0-10	8	9	9
Baseline FSFI total score, 0-36	12.4	11.1	8.3

Case 1

A 32-year-old woman with recurrent vaginismus and pelvic floor hypertonia had previously experienced partial improvement with vaginal dilator therapy, followed by symptom recurrence after discontinuing dilator use for more than one year.

During the first three treatment sessions, vulvar irritation resolved, and a progressive reduction in levator ani and puborectalis contracture was observed. The patient resumed vaginal dilator use without pain. By sessions four and five, she tolerated a size two dilator without pain, with no clinically evident transverse perineal muscle contracture. At completion of the sixth session, pelvic floor contracture was no longer evident, and she was able to insert a size five dilator easily and without pain.

At follow-up, the pain score was 0/10, and the FSFI total score had increased to 31.8/36. She reported pain-free penetrative intercourse with preserved desire, arousal, and orgasm, and no clinically evident residual pelvic floor contracture was detected.

Case 2

A 31-year-old woman presented with vaginismus and pelvic floor hypertonia in the context of marked fear and anticipatory anxiety toward vaginal penetration. Previous pelvic floor rehabilitation had been intermittently interrupted, and adherence to vaginal dilator therapy had been limited.

Following completion of the six-session radiofrequency protocol, palpation of the levator ani and transverse perineal muscles was pain-free, with no clinically evident residual contracture. She tolerated insertion of a large vaginal dilator without pain and subsequently resumed penetrative sexual activity.

At follow-up, the pain score was 0/10, and the FSFI total score had increased to 28.9/36. She reported satisfactory, pleasurable, pain-free penetrative intercourse and subsequently tolerated transvaginal ultrasonography without discomfort.

Case 3

A 45-year-old woman presented with pelvic floor hypertonia, vaginismus, and severe deep dyspareunia in the context of biopsy-confirmed vulvar lichen sclerosus and longstanding vulvovaginal symptoms. Previous treatment with high-potency topical corticosteroids, vaginal dilators, and pelvic floor physical therapy had provided incomplete symptomatic improvement.

After completion of the six-session C200 protocol, clinically evident hypertonia of the levator ani and transverse perineal muscles was no longer detected, with marked improvement in penetration-related pain. She resumed satisfactory penetrative intercourse without pain. At the post-treatment assessment, the pain score had decreased to 1/10, and the FSFI total score had increased to 29.2/36.

After completion of the radiofrequency protocol, she subsequently underwent fractional carbon dioxide laser and platelet-rich plasma treatment for persistent vulvovaginal manifestations associated with lichen sclerosus. Outcomes occurring after initiation of these interventions were not attributed to radiofrequency.

Summary of quantitative outcomes

Table 2 summarizes the quantitative evolution of the three patients treated with the C200 protocol.

**Table 2:** Individual clinical outcomes following the six-session C200 radiofrequency protocol VAS: Visual Analog Scale; FSFI: Female Sexual Function Index.

Patient	Baseline VAS (0-10)	Follow-up VAS (0-10)	Baseline FSFI total score (0-36)	Follow-up FSFI total score (0-36)	Functional outcome
Case 1	8	0	12.4	31.8	Resolution of clinically evident pelvic floor contracture; pain-free vaginal penetration and intercourse
Case 2	9	0	11.1	28.9	No residual clinically evident pelvic floor contracture; satisfactory pain-free vaginal penetration
Case 3	9	1	8.3	29.2	Resolution of clinically evident pelvic floor hypertonia; marked improvement in dyspareunia and resumption of pain-free intercourse

## Discussion

This prospective case series describes the clinical course of three women with pelvic floor hypertonia and vaginismus treated with a six-session monopolar capacitive-resistive radiofrequency protocol. Pain scores decreased in all three patients, accompanied by improvement in FSFI scores, normalization or marked reduction of clinically evident pelvic floor muscle hypertonia, and recovery of pain-free vaginal penetration. Although the magnitude and consistency of these changes are clinically noteworthy, the uncontrolled design and very small sample size preclude attribution of the observed improvements solely to the radiofrequency intervention.

The findings are broadly consistent with an emerging body of evidence suggesting that radiofrequency-based therapies may have a role in the management of myofascial pelvic pain and pelvic floor hypertonia. In a randomized pilot trial, Ashtiani et al. compared six sessions of radiofrequency modulation therapy with a tailored physiotherapy program in women with myofascial pelvic pain syndrome and found reductions in pain and improvements in pelvic floor function in both groups, with broadly comparable clinical effectiveness [4]. More recently, Li et al. compared radiofrequency therapy with manual pelvic fascial release in 176 women with myofascial pelvic pain and reported significant pain reduction with both interventions, although objective measures of pelvic floor muscle strength and surface electromyography did not differ substantially between groups [5]. These observations support the potential clinical relevance of radiofrequency while also illustrating that improvement in pain does not necessarily imply measurable restoration of pelvic floor muscle function.

More compelling evidence has subsequently emerged from a multicenter randomized placebo-controlled trial of capacitive-resistive electric transfer in women with chronic primary pelvic pain syndrome and objectively documented pelvic floor hypertonia [3]. Active treatment was associated with greater reductions in pain than sham therapy and with decreases in resting pelvic floor electromyographic activity, with improvements maintained at six-week follow-up [3]. In addition, a recent case-control study of 138 women with chronic pelvic pain and pelvic floor muscle hypertonicity found greater pain reduction when TECAR (transfer of energy capacitive and resistive) therapy was added to multimodal pelvic floor rehabilitation than with standard rehabilitation alone [6]. These studies strengthen the biological and clinical rationale for energy-transfer techniques in pelvic floor hypertonicity, although important differences in devices, treatment parameters, concomitant therapies, patient populations, and outcome assessment limit direct comparison with the present series.

Several mechanisms could plausibly contribute to the observed clinical response. Capacitive-resistive radiofrequency produces controlled tissue heating that may increase local perfusion and alter the mechanical and metabolic environment of hypertonic muscle. Thermal stimulation may also facilitate muscle relaxation and influence peripheral nociceptive signaling. However, proposed effects on microcirculation, connective tissue remodeling, inflammatory pathways, or pain neuromodulation remain incompletely characterized in the female pelvic floor. Accordingly, mechanistic explanations should be considered biologically plausible rather than established mechanisms of action for the clinical improvements observed in this series.

The three cases also illustrate the heterogeneity of patients presenting with pelvic floor hypertonia and penetration-related pain. The first patient had recurrent symptoms after discontinuation of vaginal dilator therapy, whereas the second had prominent fear and anticipatory anxiety together with interrupted adherence to pelvic floor rehabilitation. The third patient represented a substantially more complex phenotype, with biopsy-confirmed vulvar lichen sclerosus, severe dyspareunia, and structural vulvar changes in addition to pelvic floor hypertonia. Improvement in muscle tenderness and penetration was observed across these differing clinical contexts, suggesting that pelvic floor hypertonia may constitute a modifiable component of symptoms even when additional biological or psychosexual factors coexist. Nevertheless, the contribution of these comorbidities and previous or concurrent interventions cannot be separated from the effect of radiofrequency in an uncontrolled case series.

The improvement in FSFI scores observed in all three patients should also be interpreted cautiously. Sexual function is multidimensional and is influenced not only by pelvic floor pain but also by desire, arousal, lubrication, relationship factors, psychological state, and expectations regarding penetration. A reduction in pain may therefore facilitate broader improvements in sexual function without implying a direct effect of radiofrequency on each FSFI domain. Because domain-specific scores were not analyzed separately and no untreated comparator was available, the present results support an association between clinical improvement and higher overall FSFI scores rather than a direct treatment effect on global sexual function.

The third case deserves particular consideration because additional treatment with fractional carbon dioxide laser and platelet-rich plasma was initiated after completion of the C200 protocol. Outcomes documented after these interventions cannot be attributed specifically to radiofrequency. For this reason, the present analysis confines interpretation of the radiofrequency response to changes observed at completion of the six-session protocol and does not use subsequent improvement in vulvovaginal trophism or tissue characteristics as evidence of a radiofrequency effect.

The present findings therefore support further investigation rather than immediate incorporation of monopolar capacitive-resistive radiofrequency as a standard treatment for vaginismus or pelvic floor hypertonia. Future studies should use adequately powered controlled designs, clearly defined diagnostic criteria, standardized radiofrequency parameters, prespecified follow-up intervals, and objective measures of pelvic floor muscle activity in addition to patient-reported outcomes. Comparative studies against pelvic floor physical therapy or sham treatment would be particularly informative, as would assessment of whether radiofrequency provides incremental benefit when incorporated into multidisciplinary rehabilitation rather than used as a stand-alone intervention.

Limitations

This study has several important limitations. First, the sample consisted of only three patients and lacked a control or sham-treated group, precluding causal inference regarding the observed clinical improvements. The magnitude of the changes in pain and sexual function should therefore be interpreted as descriptive and hypothesis-generating.Placebo effects, regression to the mean, natural symptom fluctuation, and increased clinical attention may have contributed to the observed outcomes.

Second, pelvic floor hypertonia and its evolution were assessed primarily by clinical examination rather than by standardized objective measures such as surface electromyography, manometry, or validated quantitative muscle-tone assessment. The absence of blinded outcome assessment also introduces the possibility of observer bias. Although the VAS and FSFI provided standardized patient-reported measures, sexual function is multidimensional and may have been influenced by factors other than changes in pelvic floor muscle tone.

Third, the three patients represented clinically heterogeneous presentations, including recurrent vaginismus, relevant psychosexual and psychiatric comorbidity, and vulvar lichen sclerosus. In Case 1, resumption of vaginal dilator therapy during the treatment period represents a relevant co-intervention that may have contributed to the observed improvement. In the third case, fractional carbon dioxide laser and platelet-rich plasma were administered after completion of the radiofrequency protocol; consequently, subsequent changes in vulvovaginal trophism or other clinical outcomes cannot be attributed specifically to radiofrequency. Detailed session-level radiofrequency parameters were not systematically recorded, limiting protocol reproducibility and precluding exploration of potential dose-response relationships.

Finally, follow-up was limited and was not performed at uniformly predefined time points across all patients, restricting assessment of the durability of the observed response. Treatment parameters and clinical assessment procedures also require greater standardization to facilitate reproducibility and comparison across studies. Larger prospective controlled studies with predefined follow-up intervals, standardized diagnostic and treatment protocols, objective pelvic floor measurements, blinded assessment where feasible, and systematic adverse-event reporting are needed to determine the efficacy, durability, and safety of monopolar capacitive-resistive radiofrequency in this clinical setting.

## Conclusions

In this prospective case series of three women with pelvic floor hypertonia and vaginismus, a six-session protocol of monopolar capacitive-resistive radiofrequency was associated with substantial improvements in pelvic pain, sexual function, pelvic floor examination findings, and the ability to achieve pain-free vaginal penetration. Improvement was observed across clinically heterogeneous presentations, including patients with previous incomplete response or limited adherence to conventional management.

Given the small sample size, absence of a control group, clinical heterogeneity, and potential influence of previous or concomitant interventions, these findings cannot establish the efficacy of radiofrequency or support its routine use as a standard treatment. They should instead be considered preliminary and hypothesis-generating. Larger controlled prospective studies incorporating standardized treatment parameters, objective pelvic floor assessments, systematic safety evaluation, and predefined long-term follow-up are required to determine whether monopolar capacitive-resistive radiofrequency provides clinically meaningful and durable benefit in women with pelvic floor hypertonia and penetration-related pain.
